# Hepatic steatosis in women with polycystic ovary syndrome

**DOI:** 10.1186/s12902-023-01456-6

**Published:** 2023-09-26

**Authors:** Xinyu Hong, Zaixin Guo, Qi Yu

**Affiliations:** Department of Obstetrics and Gynecology, Peking Union Medical College Hospital, National Clinical Research Center for Obstetric & Gynecologic Diseases, Chinese Academy of Medical Sciences & Peking Union Medical College, Beijing, China

**Keywords:** Polycystic ovary syndrome, Metabolic dysfunction-associated fatty liver disease, Nonalcoholic fatty liver disease, Hepatic steatosis, Insulin resistance

## Abstract

**Background:**

This multi-center, cross-sectional study intended to explore the prevalence and risk factors of nonalcoholic fatty liver disease (NAFLD) and metabolic dysfunction-associated fatty liver disease (MAFLD) in patients with polycystic ovary syndrome (PCOS).

**Methods:**

Patients who met the PCOS Rotterdam diagnostic criteria were enrolled in 6 centers in China, and age-matched healthy volunteers were also recruited. Data were collected including medical history, physical characteristics, and blood tests (liver function, blood lipids, blood glucose and insulin, sex hormones, etc.). Transvaginal or transrectal ultrasound was employed to identify polycystic ovarian morphology (PCOM). The serological score Liver Fat Score (LFS) >-0.640 was used for the diagnosis of NAFLD, and the diagnosis of MAFLD was made according to the 2020 new definition.

**Results:**

A total of 217 PCOS patients and 72 healthy controls were included. PCOS patients had impaired glucose and lipid metabolism, higher liver enzymes and LFS. Both NAFLD (33.6%) and MAFLD (42.8%) was more prevalent in PCOS patients than in controls (4.2%, *P* < 0.001). Logistic regression results showed that HOMA-IR ≥ 3.54 and ALT ≥ 18.2 were independently associated with NAFLD (*P* < 0.001) and MAFLD (*P* ≤ 0.001). The prevalence of NAFLD was significantly higher in PCOS patients with free androgen index (FAI) > 8 (53.8% versus 17.4%, *P* < 0.001) and BMI ≥ 24 kg/m^2^ (57.3%, 11.3%, *P* < 0.001).

**Conclusion:**

The prevalence of NAFLD/MAFLD in PCOS patients was significantly higher than that in healthy controls and was independently associated with HOMA-IR and ALT. PCOS patients with overweight and elevated FAI have a higher prevalence of fatty liver.

**Supplementary Information:**

The online version contains supplementary material available at 10.1186/s12902-023-01456-6.

## Introduction

Polycystic ovary syndrome (PCOS) is the most common endocrine disease in women of childbearing age, with a prevalence of about 8–13% [1, 2]. It not only involves the reproductive system, leading to hyperandrogen, menstrual disorders and infertility, but also brings increased metabolic risk. Nonalcoholic fatty liver disease (NAFLD) is one of the representative metabolic complications, which can be accompanied by varying degrees of inflammation and fibrosis, and progress to liver cirrhosis or even liver cancer in the later stage [3]. The classical definition of NAFLD refers to steatosis not caused by alcohol or other known factors (such as viruses and drugs, etc.). The prevalence of NAFLD in PCOS patients is about 34-70%, which is much higher than that in general population (14-34%) [4]. Even after adjusting BMI (body mass index), the prevalence of NAFLD in PCOS is still 2.2–4.3 times higher than that in control [5]. The underlying mechanism of the comorbidity of PCOS and fatty liver mainly attributes to insulin resistance (IR) and hyperandrogenism. It also involves abnormal glycometabolism and dyslipidemia, obesity and chronic inflammation [4, 6–8].

Investigators reported varied risk factors for NAFLD in PCOS patients using different evaluation tools. A small-scale study involving only 29 patients and 22 healthy controls showed that hyperandrogen was significantly associated with increased liver fat assessed by ^1^H-Magnetic Resonance Spectroscopy (^1^H-MRS), the imaging gold standard for NAFLD, which is accurate, quantitative but expensive and rare [9]. Quantitative ultrasound has similar situation with 1 H-MRS, and has a higher rate of measurement failure [10]. B-mode ultrasound is the most commonly used screening method and was adopted by most studies [11], but it is extremely insensitive to mild hepatic steatosis and in obese patients [12, 13]. Risk factors were reported including androgen, IR, BMI, ALT, hsCRP (hypersensitivity C reactive protein), and TG (triglyceride), etc. [11, 14–18]. Serological scores are newly-emerged, effective and convenient non-imaging tools. A study published in 2016 involving 600 PCOS patients using liver fat score (LFS) found that LFS was significantly associated with homeostasis model assessment of insulin resistance (HOMA-IR) and lipid accumulation product (LAP), but not with free androgen index (FAI) [19]. In 2017, a study using another serological score called hepatic steatosis index (HSI) as a diagnostic method for NAFLD, including 202 PCOS patients without diabetes, concluded that hepatic steatosis was significantly associated with waist circumference and insulin resistance, but not with FAI [20].

A new definition of hepatic steatosis called metabolic dysfunction-associated fatty liver disease (MAFLD) was proposed in 2020 [21]. According to this positive diagnostic criteria, MAFLD can be diagnosed when imaging or serological or histological evidence of fatty liver disease was present and one of the following 3 criteria was met: overweight/obesity, type 2 diabetes mellitus, or metabolic disorders, regardless of alcohol consumption. After the definition of MAFLD updated, there have not been studies designed for risk factors of MAFLD in PCOS so far.

This study intended to conduct a multi-center, cross-sectional study to investigate the prevalence of NAFLD and newly defined MAFLD in PCOS patients as well as in healthy controls, to explore the risk factors of PCOS combined with NAFLD/MAFLD.

## Methods

### Subjects

Patients newly diagnosed with PCOS were consecutively collected from 6 gynecological endocrinology clinics in China. The diagnostic criteria of PCOS was the revised 2003 Rotterdam Consensus [22]. At least two of three criteria should be met: (1) oligomenorrhoea or anovulation; (2) biochemical signs of hyperandrogenemia; (3) polycystic ovarian morphology (PCOM). PCOM was defined as 12 or more follicles with a diameter of 2 to 9 mm on unilateral ovary and/or an increase in ovarian volume (> 10 mL) on ultrasound. The diagnosis of PCOS required further exclusion of other diseases that may cause hyperandrogen or abnormal ovulation, such as non-classical 21-hydroxylase deficiency, hyperprolactinemia, Cushing’s disease, untreated hypothyroidism and androgen secreting tumors [22].

Healthy volunteers were recruited in the community in Beijing, China, who were required to be female aged 18 to 40 years old with regular menstrual cycles between 21 and 35 days. Exclusion criteria were as follows: (1) current pregnancy or lactation; (2) previously diagnosed PCOS; (3) hyperandrogenic manifestations such as obvious hirsutism, acne or alopecia; (4) alcohol intake > 20 g/d; (5) previous or current viral hepatitis, autoimmune hepatitis, hereditary hepatitis, drug-induced hepatitis and other liver diseases. Examinations were performed to eliminate the potential diagnosis of PCOS and other diseases that might cause high androgen or abnormal ovulation.

Neither the PCOS patients nor the control group had been treated with medications concerning lipid-lowering, antidiabetes, antiandrogen, or estrogens during the 3 months before enrolling.

### Data collection

For both the PCOS patients and the healthy controls, medical history was collected including history of menstruation, pregnancy, gynecological diseases, other chronic diseases especially liver-related, recent medications, smoking and alcohol drinking. Physical data including height, weight, waist and hip circumference and blood pressure was recorded. Waist circumference was defined by the International Diabetes Federation (IDF) as the circumference of the midpoint line between the lowest point of the rib and the upper margin of the iliac muscle at the end of expiration, and hip circumference was defined as the maximum circumference of the hip. Blood pressure was measured by electronic sphygmomanometer in sitting position at rest. Modified Ferriman-Gallwey (mFG) index was used to evaluate the hair of 9 parts including upper lip, jaw, chest, upper abdomen, lower abdomen, arms, legs, upper back and lower back [23], and a score ≥ 5 was defined as hirsutism [24]. The Investigator Global Assessment (IGA) was used to evaluate acne of 3 parts including the facial, chest, and back regions, and a score ≥ 2 was used as the clinical standard for hyperandrogen. Hair loss was assessed using the Ludwig score. All the data were evaluated by experienced clinical staff in accordance with the uniform standards.

Blood samples were collected in the morning after 12 h of overnight fasting, during the 2nd to the 6th days of the menstrual cycle (early follicular phase). All the tests were strictly controlled according to the uniform laboratory standards, involving serum aspartate (AST), alanine aminotransferases (ALT), γ-glutamyltransaminase(GGT), alkaline phosphatase (ALP), total bilirubin (TBil); fasting and 2 h postprandial glucose (0hGlu, 2hGlu) and insulin (0hINS, 2hINS); total cholesterol (TC), high-density lipoprotein (HDL), low-density lipoprotein (LDL), triglycerides (TGs); hypersensitivity C reactive protein (hsCRP); follicle stimulating hormone (FSH), luteinizing hormone (LH), prolactin (PRL), total testosterone (T), sex hormone-binding protein (SHBG), anti-müllerian hormone (AMH); free triiodothyronine (FT3), free thyroxine (FT4), and thyroid stimulating hormone (TSH). Insulin and thyroid function were determined by direct chemiluminescence using immunoassay system Atellica and matching kits (Siemens, Germany); sex hormones were analyzed by chemiluminescence using DXI800 automatic chemiluminescence analyzer and matching kits (Beckman, USA). For postprandial glucose and insulin, 75 g anhydrous glucose was prepared with 300 mL water and was consumed within 5 min.

We further calculated several variables using the following formulas [25–29]:

$$\mathrm{Lipid}\;\mathrm{accumulation}\;\mathrm{product}\;(\mathrm{LAP})=\;\left[\mathrm{Waist}\;\mathrm{Circumstance}\left(\mathrm{cm}\right)-58\right]\times\mathrm{TG}\left(\mathrm{mmol}/\mathrm L\right)$$

$$\mathrm{FAI}=\left[\mathrm T\left(\mathrm{nmol}/\mathrm L\right)\times100\right]/\mathrm{SHBG}\left(\mathrm{nmol}/\mathrm L\right)$$

$$\mathrm{HOMA}-\mathrm{IR}=0\mathrm{hINS}\;\left(\mathrm{mIU}/\mathrm L\right)\times0\mathrm{hGlu}\;\left(\mathrm{mmol}/\mathrm L\right)/22.5$$

$$\mathrm{Quantitative}\;\mathrm{insulin}\;\mathrm{sensitivity}\;\mathrm{check}\;\mathrm{index}\;(\mathrm{QUICKI})=1/\left[\left[\mathrm{Log}\;\lbrack0\mathrm{hGlu}\;(\mathrm{mg}/\mathrm{dL})\right]+\mathrm{Log}\;\left[0\mathrm{hINS}\;(\mathrm{mIU}/\mathrm L)\right]\right]$$

$$\mathrm{Gutt}\;\mathrm{index}=75000\left(\mathrm{mg}\right)+\left[\left(0\mathrm{hGlu}-2\mathrm{hGlu}\right)\left(\mathrm{mg}/\mathrm L\right)\times0.19\times\mathrm{Weight}(\mathrm{kg})\right]/\left[120\times{\mathrm{Glu}}_{\mathrm{mean}}\left(0\mathrm h,2\mathrm h\right)\left(\mathrm{mmol}/\mathrm L\right)\times\mathrm{Log}\left[{\mathrm{INS}}_{\mathrm{mean}}\left(0\mathrm h,2\mathrm h\right)\left(\mathrm{mIU}/\mathrm L\right)\right]\right]$$

From the 2nd to the 7th day of the menstrual cycle, the subjects were examined by transvaginal or transrectal B-mode ultrasound. The ovarian volume (mL) was defined as 0.5× length (cm) × width (cm) × thickness (cm), or 0.5× length (cm) × width^2^ (cm^2^) when the thickness was not measured. The number and size of bilateral follicles were also recorded. All the data were measured by experienced B-ultrasound doctors. The center frequency of B-ultrasound was 5.0-7.5 MHz, and the probe frequency was 3.3–7.5 MHz.

### Outcomes

The NAFLD Liver fat score (LFS), a serological score based on ^1^H-MRS, was calculated, and a value of LFS>-0.640 was considered to be the diagnosis of NAFLD [5, 30]. Neither the PCOS patients nor the healthy volunteers matched the standard of AFLD in this study. MAFLD was defined according to the 2020 definition [21].

LFS = -2.89 + 1.18 × MetS (yes = 1 / no = 0) + 0.45 × T2DM (yes = 2 / no = 0) + 0.15 × 0hINS (mIU/L) + 0.04 × AST (U/L)- 0.94 × AST/ALT. MetS was defined as metabolic syndrome based on the 2005 IDF diagnostic criteria. T2DM was defined as type 2 diabetes mellitus according to the WHO criteria.

Hepatic steatosis index (HSI) [31] and fatty liver index (FLI) [32] were also calculated and compared with LFS .

HSI = 8× ALT/AST + BMI (kg/m^2^) + 2 (if DM) + 2 (if female). Hepatic steatosis was diagnosed when HSI > 36, and excluded when HSI < 30.

FLI = [e^M^/ (1 + e^M^)]× 100, M = 0.953 × ln [TG (mg/dL)] + 0.139 × BMI (kg/m^2^) + 0.718 × ln [GGT (U/L)] + 0.053 × WC (cm) -15.745. Hepatic steatosis was diagnosed when FLI ≥ 60, and excluded when FLI < 30.

### Statistical analysis

Statistical analysis was performed using IBM SPSS Statistics 25.0 and MedCalc 19.6.4. For quantitative data, t test or Mann-Whitney U test was used for comparison between 2 groups. Comparisons among multiple groups were performed using ANOVA or Kruskal-Wallis H test. For categorical data, Chi-square test or Fisher exact probability test was used for comparison between groups. And for ranked data, Mann-Whitney U test and Kruskal-Wallis H test for comparison. Analysis of covariance (ANCOVA) was used to adjust the BMI mismatch between the PCOS group and the control group.

For continuous variables, Spearman’s correlation coefficient (r) was used for the correlation between different variables, and *r* > 0.300 was considered to be significant. Multivariate binary logistic regression analysis was performed on all variables that were significantly associated with NAFLD/MAFLD-LFS (*P* < 0.05). Receiver operating characteristic curve (ROC) and the area under the curve (AUC) were used to determine the best cut-off value for continuous variables to be converted into categorical variables. *P*-values < 0.05 were considered statistically significant.

## Results

We collected 492 patients diagnosed as PCOS from July 2019 to April 2022. After excluding those who could not strictly constitute the Rotterdam criteria and those who could not calculate LFS due to missing data, a total of 217 patients were included. Seventy-nine healthy volunteers were recruited in Beijing, China from March 2022 to May 2022. Among them, 5 cases were excluded for not completing all the examinations, and 2 cases were diagnosed as PCOS for PCOM and hirsutism. Finally, 72 healthy controls were included.

### Anthropometric and biochemical characteristics of PCOS and controls

As shown in Table 1, PCOS patients and healthy controls were age matched (28.4 and 27.7 years, *P* = 0.200). PCOS women had significantly higher BMI, waist circumference, waist-hip ratio and blood pressure; higher blood glucose, insulin and more insulin resistance assessed by HOMA-IR, QUICKI and Gutt index; higher TC, TG, LDL-c, LAP and lower HDL-c; as well as higher hsCRP, which can be regarded as a cardiometabolic risk marker. PCOS women also had significantly more obvious hyperandrogenic signs, higher testosterone and FAI, and higher AMH. The proportion of PCOM in the PCOS group was 90.6%, which was much higher than that in the control group (48.6%).

The levels of ALT, AST, GGT and ALP in PCOS group were significantly higher than those in control group. The three serological scores of hepatic steatosis were significantly higher in the PCOS group (Table 1, all *P* < 0.001).

After adjustment for BMI between PCOS and control groups, PCOS group still had worse anthropometric data, worse conditions of glucose and lipid metabolism, and more hyperandogenic parameters. PCOS women still had higher liver enzymes and higher level of hepatic steatosis evaluated by serological scores (Table 1).

**Table 1:** Anthropometric and biochemical characteristics of PCOS and controls

	PCOS (*N* = 217)	Control (*N* = 72)	*P*	P*
**Anthropometric data**				
Age (years)	28.4 (24.4, 32.5)	27.7 (23.0, 32.4)	0.220	0.599
BMI (kg/m^2^)	23.7 (21.3, 26.5)	21.1 (19.6, 23.4)	< 0.001	-
Waist circumstance (cm)	80.0 (74.0, 89.0)	71.0 (67.3, 76.8)	< 0.001	< 0.001
Hip circumstance (cm)	97.0 (92.0, 102.0)	94.0 (90.2, 97.8)	0.002	0.036
Waist-hip ratio	0.83 (0.79, 0.88)	0.76 (0.74, 0.79)	< 0.001	< 0.001
SBP (mmHg)	120.5 (108.6, 132.4)	108.6 (99.3, 117.8)	< 0.001	< 0.001
BP (mmHg)	75.0 (70.0, 83.0)	66.0 (63.0, 71.0)	< 0.001	< 0.001
**Hyperandrogenic signs**				
mFG hair score	3 (1, 6)	1 (0, 2.75)	< 0.001	< 0.001
IGA acne score	1 (0, 3)	0 (0, 1)	< 0.001	< 0.001
Ludwig alopecia score	0 (0, 1)	0 (0, 0)	< 0.001	< 0.001
**Liver function**				
AST (U/L)	18.0 (15.0, 23.0)	15.0 (13.2, 17.8)	< 0.001	< 0.001
ALT (U/L)	17.5 (12.6, 25.4)	11.0 (9.0, 14.0)	< 0.001	< 0.001
AST/ALT	1.02 (0.80, 1.31)	1.37 (1.17, 1.63)	< 0.001	0.001
GGT (U/L)	18.0 (13.2, 24.0)	12.0 (11.0, 14.0)	< 0.001	< 0.001
ALP (U/L)	60.4 (46.9, 74.2)	50.4 (37.7, 63.1)	< 0.001	0.007
TBil (µmol/L)	10.4 (7.5, 13.2)	9.2 (7.3, 12.4)	0.487	0.039
**Glucose metabolism**				
0hGlu (mmol/L)	5.1 (4.7, 5.4)	4.8 (4.5, 5.1)	< 0.001	0.138
2hGlu (mmol/L)	6.2 (5.2, 7.2)	5.4 (4.6, 6.1)	< 0.001	0.009
0hINS (µIU/mL)	10.4 (7.1, 16.5)	6.3 (5.0, 8.1)	< 0.001	0.001
2hINS (µIU/mL)	51.9 (34.5, 91.6)	39.0 (25.6, 58.1)	< 0.001	0.733
HOMA-IR	2.24 (1.50, 3.84)	1.41 (1.03, 1.83)	< 0.001	0.002
QUICKI	0.34 (0.30, 0.37)	0.36 (0.34, 0.39)	< 0.001	0.001
Gutt index	73.0 (55.4, 87.1)	87.8 (74.7, 110.9)	< 0.001	0.004
**Lipid metabolism**				
TC (mmol/L)	4.74 (4.32, 5.22)	4.48 (3.91, 5.00)	0.004	0.044
HDL (mmol/L)	1.28 (1.10, 1.52)	1.44 (1.28, 1.71)	< 0.001	0.069
LDL (mmol/L)	2.95 (2.56, 3.44)	2.43 (2.13, 2.92)	< 0.001	0.005
TG (mmol/L)	1.00 (0.74, 1.49)	0.75 (0.56, 1.10)	< 0.001	< 0.001
LAP	22.90 (13.10, 39.84)	9.87 (5.69, 14.98)	< 0.001	< 0.001
hsCRP (mg/L)	1.39 (0.42, 2.59)	0.65 (0.26, 1.13)	0.002	0.432
**Sex hormone**				
FSH (IU/L)	6.35 (5.36, 7.53)	7.23 (6.28, 8.19)	< 0.001	0.001
LH (IU/L)	10.94 (6.57, 16.38)	4.62 (3.64, 5.88)	< 0.001	0.031
PRL (ng/mL)	12.0 (8.2, 16.9)	17.5 (13.4, 24.6)	< 0.001	< 0.001
T (ng/mL)	0.69 (0.49, 0.88)	0.48 (0.38, 0.58)	< 0.001	< 0.001
SHBG (nmol/L)	34.4 (20.3, 46.8)	48.5 (34.1, 64.9)	< 0.001	0.154
FAI	7.03 (3.69, 12.92)	3.22 (2.30, 4.87)	< 0.001	< 0.001
AMH (ng/mL)	7.99 (5.68, 11.42)	4.90 (2.91, 6.90)	< 0.001	< 0.001
**Thyroid function**				
FT3 (pg/mL)	3.41 (3.24, 3.74)	3.19 (3.00, 3.43)	0.063	0.017
FT4 (ng/dL)	1.24 (1.07, 1.42)	1.22 (1.09, 1.34)	0.291	0.565
TSH (µIU/mL)	1.93 (1.31, 3.22)	2.36 (1.79, 3.26)	0.139	0.058
**Serological scores of hepatic steatosis**				
LFS	-1.52 (-2.26, -0.14)	-2.64 (-2.96, -2.10)	< 0.001	0.002
HSI	33.8 (29.8, 39.0)	29.1 (27.3, 30.9)	< 0.001	< 0.001
FLI	17.48 (4.54, 45.97)	3.60 (2.17, 8.61)	< 0.001	0.001

The measurement data with normal distribution are expressed by mean ± standard deviation, while those with non-normal distribution are expressed by median and quartile

*BMI* Body mass index, *SBP *Systolic blood pressure, *DBP *Diastolic blood pressure, *AST *Aspartate aminotransferase, *ALT *Alanine aminotransferase, *GGT* Gamma-glutamyltransaminase, *ALP *Alkaline phosphatase, *TBil *Total bilirubin, *0hGlu, 2hGlu, 0hINS and 2hINS *Fasting and 2 h postprandial glucose and insulin, *HOMA-IR *Homeostatic model assessment of insulin resistance, *QUICKI* Quantitative insulin sensitivity check index, *TC *Total cholesterol, *HDL* High-density lipoprotein, *LDL *Low-density lipoprotein, *TG* Triglycerides, *LAP* Lipid accumulation product, *hsCRP *Hypersensitivity C reactive protein, *FSH* Follicle stimulating hormone, *LH* Luteinizing hormone, *PRL* Prolactin, *T* Total testosterone, *SHBG *Sex hormone-binding protein, *FAI* Free androgen index, *AMH *Anti-müllerian hormone, *FT3 *free triiodothyronine, *FT4 *Free thyroxine, *TSH *Thyroid stimulating hormone, *LFS *Liver fat score, *HSI *Hepatic steatosis index, *FLI *Fatty liver index

*P adjusted for age using ANCOVA

### Prevalence of NAFLD and MAFLD in PCOS and controls

Seventy-three patients (33.6%) were diagnosed with NAFLD in the PCOS group, which was significantly higher than that in the control group (4.2%, *P* < 0.001, OR = 11.660, 95%CI 3.548, 38.316). After adjustment for BMI, the prevalence of NAFLD in the two groups was 29.6% (ANCOVA 95%CI: 24.6-34.6%) and 16.8% (ANCOVA 95%CI: 8.1-25.6%, *P* = 0.015) respectively. However, among the 76 patients with NAFLD, only 23 had elevated ALT and 12 had elevated AST.

According to the definition of MAFLD, patients with missing metabolic risk factors were excluded, and 166 PCOS patients were finally included in the analysis. A total of 71 PCOS patients (42.8%) were diagnosed with MAFLD, which was significantly higher than that of the control group (4.2%, *P* < 0.001, OR = 17.189, 95%CI 5.198, 56.850).

### Risk factors associated with NAFLD/MAFLD in PCOS women

**Fig. 1:**
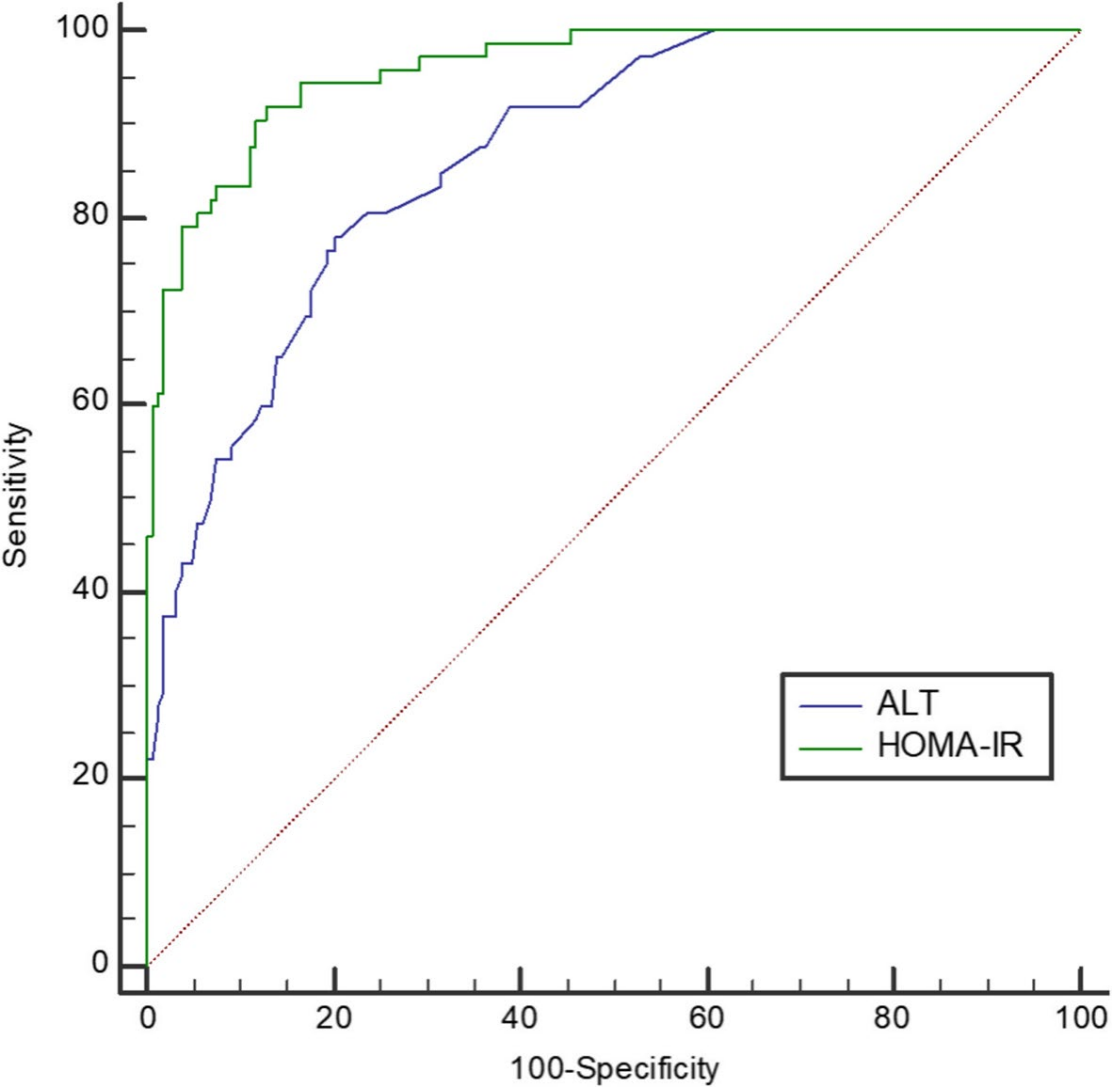
ROC curves: the predictive validity of ALT and HOMA-IR respectively for LFS

According to the correlation analysis (Supplement Table 1), LFS was associated with BMI, waist circumference, ALT, GGT, AST/ALT, hsCRP, TG, HDL, LAP, 0hGlu, 0hINS, 2hINS, HOMA-IR, SHBG and FAI (*r* > 0.300). Considering the collinearity effects, we omitted variables involved in formulas of LFS, LAP, HOMA-IR and FAI. GGT and hsCRP were excluded due to data missing. Only BMI, ALT, HDL, LAP, 2hINS, HOMA-IR and FAI finally entered binary logistic regression, which concluded that NAFLD was associated with HOMA-IR (*P* < 0.001) and ALT (*P* = 0.003), but not FAI (Table 2). According to the ROC curves (Fig. 1), both HOMA-IR and ALT had a good predictive effect on NAFLD. The AUC of HOMA-IR was 0.955 (95%CI 0.927–0.982, sensitivity 0.812, specificity 0.964), and the AUC of ALT was 0.808 (95%CI 0.748–0.867, sensitivity 0.754, specificity 0.717). Repeated logistic regression with the dichotomous variables HOMA-IR ≥ 3.54 and ALT ≥ 18.2 at their best cut-off values showed robust results in the final model (both *P* < 0.001, Table 3).

**Table 2:** Risk factors (continuous variables) associated with NAFLD (LFS > -0.640) by binary logistic regression

Continuous variables	OR (95% CI)	*P*
HOMA-IR	17.723 (4.856, 64.684)	< 0.001
ALT	1.221 (1.073, 1.391)	0.003

*ALT* Alanine aminotransferase, *HOMA-IR *Homeostatic model assessment of insulin resistance

**Table 3:** Risk factors (categorical variables) associated with NAFLD (LFS > -0.640) by binary logistic regression

Categorical variables	OR (95% CI)	*P*
HOMA-IR ≥ 3.54	450.291 (54.895, 3693.610)	< 0.001
ALT ≥ 18.2	41.417 (5.379, 318.869)	< 0.001

PCOS patients with NAFLD tended to have higher rates of metabolic disorders than those without NAFLD. Over 80% of PCOS patients with NAFLD had central obesity and insulin resistance. 74% of these patients had an elevated ALT level, but only 31.5% had liver enzymes within abnormal range. Up to 68.9% of them had a higher FAI. However, there were no difference in hirsutism, acne and PCOM between patients with and without NAFLD (Table 4).

**Table 4:** Risk factors in PCOS patients with NAFLD and without NAFLD

	Non-NAFLD N(%)	NAFLD N(%)	*P*
Central obesity	47 (35.3%)	62 (88.6%)	< 0.001
Increased blood pressure	8 (17.4%)	19 (52.8%)	0.001
Hypertriglyceridemia	13 (9.1%)	27 (37.5%)	< 0.001
Hyperlipidemia	47 (32.9%)	42 (58.3%)	< 0.001
Abnormal blood glucose	24 (16.9%)	30 (41.7%)	< 0.001
HOMA-IR ≥ 3.54	5 (3.6%)	56 (81.2%)	< 0.001
Metabolic syndrome	1 (0.7%)	35 (47.9%)	< 0.001
Abnormal liver enzymes	5 (3.5%)	23 (31.5%)	< 0.001
ALT ≥ 18.2	42 (29.2%)	54 (74.0%)	< 0.001
FAI > 8	36 (28.6%)	42 (68.9%)	< 0.001
Hirsutism	42 (30.0%)	28 (38.9%)	0.964
Acne	68 (48.2%)	31 (43.1%)	0.674
PCOM	114 (88.4%)	60 (95.2%)	0.124

The ‘N (%)’ in each cell represented ‘the number of patients with this risk factor in this age group (the percentage of patients with this risk factor in this age group). Patients were excluded if the factor to be analyzed was missing

For risk factors of MAFLD, after screening by non-parametric test and excluding variables of collinearity and missing, BMI, ALT, HDL, LAP, 2hINS, HOMA-IR and FAI finally entered logistic regression. MAFLD was significantly correlated with HOMA-IR (*P* < 0.001) and ALT (*P* = 0.002) (Table 5). The ROC curves showed that the cut-off value of HOMA-IR was still 3.54 (AUC = 0.945, 95%CI 0.911–0.979), and the cut-off value of ALT was still 18.2 (AUC = 0.808, 95%CI 0.731–0.866). Logistic regression was then repeated with HOMA-IR ≥ 3.54 and ALT ≥ 18.2, and the results were robust (Table 6).

**Table 5:** Risk factors (continuous variables) associated with MAFLD by binary logistic regression

Continuous variables	OR (95% CI)	*P*
HOMA-IR	15.647 (4.371, 56.017)	< 0.001
ALT	1.219 (1.072, 1.388)	0.003

**Table 6:** Risk factors (categorical variables) associated with MAFLD by binary logistic regression

Categorical variables	OR (95% CI)	*P*
HOMA-IR ≥ 3.54	333.945 (39.186, 2845.890)	< 0.001
ALT ≥ 18.2	37.579 (4.818, 293.124)	0.001

### Prevalence of NAFLD in PCOS patients of different phenotypes

We divided the PCOS patients into four phenotypes according to the Rotterdam criteria: phenotype A (clinical and/or biochemical hyperandrogen + oligoamenorrhea + PCOM), phenotype B (hyperandrogen + oligoamenorrhea), phenotype C (hyperandrogen + PCOM) and phenotype D (oligoamenorrhea + PCOM). Twenty-nine patients (eleven of them with NAFLD) without ultrasound data were excluded from this analysis. Phenotype A accounted for the largest proportion (134 out of 188 PCOS patients), and 18 patients of phenotype B, only 5 patients of phenotype C, and 31 patients of phenotype D. The prevalence rates of NAFLD in patients with phenotype A to D were 36.6%, 16.7%, 40.0% and 25.8% (Supplementary Table 2), without significant difference among 4 groups. The prevalence rates of NAFLD in phenotype A, C and D were significantly higher than that in control group. Prevalence rates of MAFLD showed no significance in phenotype A to D but were all significantly higher than the control group (Supplementary Table 3).

Hyperandrogenism was defined as FAI > 8, i.e. the 95th percentile of FAI in the control group in this study. The prevalence of NAFLD showed significant difference between PCOS patients with and without hyperandrogenism (53.8% vs. 17.4%, *P* < 0.001). There was no significant difference of NAFLD prevalence in PCOS patients with/without PCOM, hirsutism, acne, and alopecia (*P* > 0.05, Supplementary Table 4).

### Prevalence and risk factors of NAFLD in PCOS of different age groups

The PCOS patients were divided into three groups according to age: 18 to 25 years old (*n* = 39), 26 to 30 years old (*n* = 74) and over 30 years old (*n* = 53). There was no significant difference in the prevalence of NAFLD among the three groups (38.3%, 30.8% and 34.9%; *P* = 0.645).

Comparing the risk factors of hepatic steatosis among the three groups, the proportions of central obesity, hypertriglyceridemia and hyperlipidemia in the group over 30 years old were significantly higher than those in younger groups (*P* < 0.05). There were no significant differences in other metabolic risk factors, liver enzymes, and PCOS characteristics among the three groups (Table 7).

**Table 7:** Risk factors of NAFLD in different age groups of PCOS

	18 ~ 25 years, N(%)	26 ~ 30 years, N(%)	Over 30 years, N(%)	*P*
Central obesity	22 (50.0%)	44 (44.9%)	43 (70.5%)**	0.006
Increased blood pressure	6 (20.7%)*	9 (32.1%)	12 (48.0%)*	0.103
Hypertriglyceridemia	3 (6.7%)*	20 (18.7%)	17 (27.0%)*	0.028
Hyperlipidemia	11 (24.4%)**	45 (42.1%)	33 (52.4%)	0.014
Abnormal blood glucose	10 (22.2%)	24 (22.4%)	20 (32.3%)	0.319
HOMA-IR ≥ 3.54	15 (34.9%)	30 (28.6%)	16 (27.1%)	0.669
Metabolic syndrome	8 (17.0%)	14 (13.1%)	14 (22.2%)	0.301
Abnormal liver enzymes	8 (17.0%)	12 (11.2%)	8 (12.7%)	0.612
ALT ≥ 18.2	21 (44.7%)	48 (44.9%)	27 (42.9%)	0.966
FAI > 8	16 (40.0%)	39 (41.9%)	23 (42.6%)	0.967
Hirsutism	19 (41.3%)	35 (33.7%)	16 (25.8%)	0.234
Acne	23 (50.0%)	54 (51.9%)	22 (34.9%)*	0.088
PCOM	40 (90.9%)	84 (90.3%)	50 (90.9%)	0.990

The ‘N (%)’ in each cell represented ‘the number of patients with this risk factor in this age group (the percentage of patients with this risk factor in this age group)’. Patients were excluded if the factor to be analyzed was missing

Central obesity was defined as waist circumference ≥ 80 cm; overweight as BMI ≥ 24 kg/m^2^. Abnormal blood glucose was defined as pre-diabetes (in the definition of MAFLD, i.e. the ADA definition) or diabetes, or related drug treatment. Hyperlipidemia was defined as TG ≥ 1.7 mmol/L or TC ≥ 5.70 mmol/L or LDL-c ≥ 3.37 mmol/L or HDL-c < 0.93 mmol/L. Other definitions were described previously

The specific p values between any 2 groups were listed in Supplementary Table 5

** significant difference with other 2 groups (*p* < 0.05)

* significant difference with the other group marked with ‘*’ (*p* < 0.05)

### Prevalence and risk factors of NAFLD in overweight and non-overweight PCOS patients

Eight cases (3.7%) were excluded from this analysis due to missing BMI data. PCOS patients were divided into 103 (49.3%) overweight (BMI ≥ 24 kg/m^2^) and 106 (50.7%) non-overweight (BMI < 24 kg/m^2^) patients. The prevalence of NAFLD in the overweight group was 57.3%, significantly higher than that in the non-overweight group (11.3%, *P* < 0.001) and the control group (4.2%, *P* < 0.001). While the prevalence of NAFLD in the non-overweight group did not show the difference from that in the control group (*P* = 0.106).

All the metabolic risk factors, abnormal liver enzymes and hyperandrogenism were more common in the overweight group (all *P* < 0.05), and there were no significant differences in hyperandrogenic signs and PCOM (all *P* > 0.05, Table 8). The most common risk factor in overweight group was central obesity (90.3%), and then were ALT ≥ 18.2 U/L (59.2%), FAI > 8 (57.5%) and HOMA-IR ≥ 3.54 (50.0%). Abnormal liver-enzymes (ALT/AST//ALP/GGT) were uncommon in both groups, with odds of 19.4% and 7.5%, respectively.

**Table 8:** Risk factors of NAFLD in overweight and non-overweight women with PCOS

	Non-overweight group N(%)	Overweight group N(%)	*P*
Central obesity	16 (16.0%)	93 (90.3%)	< 0.001
Increased blood pressure	3 (8.8%)	24 (50.0%)	< 0.001
Hypertriglyceridemia	13 (12.5%)	27 (26.2%)	0.012
Hyperlipidemia	36 (34.6%)	50 (48.5%)	0.042
Abnormal blood glucose	18 (17.1%)	36 (35.6%)	0.003
HOMA-IR ≥ 3.54	10 (9.9%)	59 (50.0%)	< 0.001
Metabolic syndrome	1 (0.9%)	35 (34%)	< 0.001
Abnormal liver enzymes	8 (7.5%)	20 (19.4%)	0.012
ALT ≥ 18.2	32 (30.2%)	61 (59.2%)	< 0.001
FAI > 8	25 (26.6%)	50 (57.5%)	< 0.001
Hirsutism	34 (32.4%)	33 (32.7%)	0.964
Acne	50 (47.6%)	45 (44.1%)	0.613
PCOM	84 (88.4%)	82 (92.1%)	0.397

The ‘N (%)’ in each cell represented ‘the number of patients with this risk factor in this age group (the percentage of patients with this risk factor in this age group). Patients were excluded if the factor to be analyzed was missing

In overweight patients, after preliminary screening by non-parametric tests, BMI, ALT, HDL-c, LAP, 2hINS, HOMA-IR and FAI finally entered in the binary logistic regression. Three variables including HOMA-IR (*P* = 0.009), ALT (*P* = 0.036) and LAP (*P* = 0.057) (Supplementary Table 6). The cut-off values of the three variables were 3.21 (AUC = 0.956, 95%CI 0.918–0.994), 19.2 (AUC = 0.758, 95%CI 0.659–0.858) and 41.43 (AUC = 0.752, 95%CI 0.659–0.858), respectively. Repeated logistic regression analysis with the dichotomous variables showed that HOMA-IR ≥ 3.21 (*P* < 0.001) and LAP ≥ 41.43 (*P* = 0.013) were risk factors for NAFLD in overweight PCOS patients (Supplementary Table 7).

The same method was used in non-overweight patients. Considering the small sample size in this group (only 12 cases with NAFLD), only ALT, LAP and HOMA-IR were included in the binary logistic regression model. HOMA-IR (*P* = 0.079) and ALT (*P* = 0.067) were finally included in the logistic model (Supplementary Table 8). The cut-off values of the two variables were 2.88 (AUC = 0.938, 95%CI 0.869-1.000) and 18.4 (AUC = 0.813, 95%CI 0.685–0.940), respectively. Repeated logistic regression showed HOMA-IR ≥ 2.88 (*P* < 0.001) and ALT ≥ 18.4 (*P* = 0.026) were independent risk factors for NAFLD in non-overweight PCOS patients (Supplementary Table 9).

### Comparison of the three hepatic steatosis assessment scores

HSI score and FLI score could be calculated in 209 and 83 patients respectively, and in all the 72 controls. Eighty-one PCOS patients (38.8%) and 6 controls (8.3%) were diagnosed as NAFLD-HSI, while 53 (25.3%) patients and 46 controls (63.9%) were excluded. Fourteen PCOS patients (16.9%) and 1 control (1.4%) were diagnosed as NAFLD-FLI, and 49 (59.0%) patients and 69 controls (95.8%) were excluded. The correlation analysis of the three serological scores showed that LFS was moderately correlated with HSI (*r* = 0.769) and FLI (*r* = 0.768), while HSI was highly correlated with FLI (*r* = 0.895).

## Discussion

Hepatic steatosis is prevalent in women with PCOS. In this study, the prevalence of NAFLD in PCOS patients was 33.5%, comparing with only 4.2% in healthy controls. After adjustment for BMI, there was still significant difference in the prevalence between two groups. The prevalence of NAFLD in this study was slightly lower than the previously reported 34-70% in PCOS patients and 14-34% in healthy controls [4], possibly due to ethnicity and region. A study on the prevalence of NAFLD in the Asia-Pacific region pointed out that the prevalence of NAFLD in the general population in China was estimated to be 5-24% [33], which was lower than that in European and American populations. The prevalence of MAFLD was 42.8%, which was significantly higher than that of the control group (4.2%, *P* < 0.001). The definition of MAFLD is more complicated than that of NAFLD, thus it requires much more elements to make a diagnosis or to complete a study. A specific prospective study for MAFLD is needed to draw a more realistic conclusion.

The LFS serological score was finally selected as the diagnostic standard of NAFLD in this study after cautious comparison. At present, liver histological diagnosis, imaging, and serum biomarkers/scores were considered as the main diagnostic methods [12, 13]. Liver biopsy is an invasive procedure and is not generally performed in suspected NAFLD women of PCOS. ^1^H-MRS is the gold standard for imaging diagnosis by measuring MRI-PDFF ≥ 5.56% [34]. However, studies using ^1^H-MRS are few and generally have a small size due to its high cost and unavailable of the facilities [9, 12]. Controlled attenuation parameter (CAP) measured by quantitative ultrasound also has strong objectivity and accuracy, but it also requires a complicate devices and has a high measurement failure rate [10]. Ultrasound is the most common screening method for hepatic steatosis with low cost, but is not sensitive to mild steatosis and advanced fibrosis, not quantified and is greatly affected by the abdominal fat thickness and the techniques of operators [13]. A variety of serological scores for liver steatosis have been constructed, including LFS, FLI, HSI and NAFLD Ridge score etc., the AUC of those in external verification is all above 0.80 [3]. LFS [30] and NAFLD Ridge score [35] were established based on the gold imaging standard ^1^H-MRS, while HSI [31] and FLI [32] were established based on ultrasound. Comparing LFS and NAFLD Ridge score, the former one has more available elements. Therefore, we finally chose LFS as the diagnostic tool for fatty liver in this study. Results of this study showed that both HSI and FLI were moderately correlated with LFS, and HSI was highly correlated with FLI. This could be explained that LFS was based on ^1^H-MRS, while HSI and FLI are based on ultrasound, which further supported the accuracy of LFS.

NAFLD was significantly related with elevated HOMA-IR and ALT in this study, but not with FAI and other androgen indicators. Insulin resistance and hyperandrogenism have been regarded as core factors of NAFLD in PCOS with evidence of both animal experiments and human researches. Although FAI was not finally included in the prediction model in our study, PCOS patients with hyperandrogenism showed a much higher prevalence of NAFLD (53.8% vs. 17.4%, *P* < 0.001), which indicated hyperandrogenism might lead to NAFLD by acting on insulin resistance as an intermediate. Another factor, ALT, is often used to reflect liver inflammation and injury in NAFLD patients. Despite the high prevalence of NAFLD in the PCOS patients, only 26 (12.0%) of all PCOS patients in this study had abnormally elevated ALT (> 40 U/L), and only 23 (30.3%) of the 76 patients with both PCOS and NAFLD had abnormally elevated ALT. A large study involving 18,825 persons showed that ALT abnormalities accounted for only 2% in the general female population, 5% in patients with type 2 diabetes, and 7% in obese women [36]. A meta-analysis showed that up to 25% of NAFLD patients and 19% of NASH patients had normal ALT [37]. Abnormal ALT elevation is not common, and cannot effectively identify the risk of NAFLD in PCOS patients at an early stage. In our study, ALT ≥ 18.2 U/L was proved to be independently related to NAFLD, which indicated that elevated ALT within the normal range should also be of concern, especially in high-risk patients such as those who are overweight. However, according to the risk prediction model obtained by binary logistic regression, the OR value of ALT was quite small compared with HOMA-IR. Therefore, ALT must be combined with metabolic indicators (especially HOMA-IR) to comprehensively judge the overall risk of liver steatosis. On the other hand, patients in our study were mostly in the early stage of NAFLD with unobvious elevation of ALT, which may lead to this lower cut-off value within normal range.

The trend existed that all phenotypes of PCOS patients tended to have higher prevalence of hepatic steatosis than the healthy controls, and showed no difference between phenotypes. The prevalence of NAFLD between phenotype B and the control group (*P* = 0.092), which seemed inconsistent with the evidence in previous studies [9, 11, 16, 38]. Besides, the number of phenotype A (*N* = 134 out of 188) was far more than that of phenotype B (*N* = 18) and C (*N* = 5) in this study. Actually, in our clinical practice, most patients came to the clinic with their main complaints of menstrual disorders, and patients with hyperandrogen and PCOM often accompanied with oligoamenorrhea. And some patients were excluded due to the missing ultrasound data (the diagnosis of PCOS could be made without B ultrasound) in this retrospective study, which could also be a possible reason.

Overweight patients had a much higher prevalence of NAFLD than non-overweight patients and the controls (*P* < 0.001), while non-overweight patients and healthy controls showed similar prevalence of NAFLD (*P* = 0.106). Therefore, more attention should be paid to overweight PCOS patients, and the importance of weight control should be emphasized. By logistic regression, HOMA-IR and LAP (involving waist circumference and triglycerides) were finally included in the overweight group, while HOMA-IR and ALT were included in the non-overweight group. Previous literature showed that LAP performed best in people with hypertriglyceridemia (AUC 0.73) [3, 16], which may be the explanation for the difference between the two groups, that is, the proportion of hypertriglyceridemia was higher in the overweight group (*P* < 0.001).

Our study had limitations. The sample size was relatively small, although had reached the expected sample size for statistical analysis, there was no validation and no more subgroup analyses in different phenotypes of PCOS. The impact of data missing in the variables involved in MAFLD could not be ignored due to its retrospective design before the definition of MAFLD came out in 2020. In the future, larger prospective studies with follow-up plans should be carried out to evaluate the prevalence and risk factors of NAFLD/MAFLD in different subgroups of PCOS.

## Conclusion

The prevalence of NAFLD defined by serological scores in PCOS patients was significantly higher than that in heathy controls in this study, even after adjustment for BMI. The prevalence of MAFLD also showed significant difference between the PCOS patients and the healthy controls. NAFLD/MAFLD was independently associated with elevated HOMA-IR (≥ 3.54) and ALT (≥ 18.2 U/L). More attention should be paid to elevated ALT within the normal range, especially in patients with obvious risk factors such as insulin resistance. PCOS patients with overweight and elevated FAI have a much higher prevalence of fatty liver, indicating the importance of weight control.

### Supplementary Information

**Additional file 1.**

## Data Availability

All data generated or analysed during this study are included in this published article.
