# Supplementary material for: Hepatic steatosis in women with polycystic ovary syndrome
Source: BMC Endocr Disord. 2023 Sep 26;23:207. doi: 10.1186/s12902-023-01456-6 (PMC10521461; doi:10.1186/s12902-023-01456-6)
Supplement: Supplementary file 1 — Additional file 1. [file 12902_2023_1456_MOESM1_ESM.docx]

**Supplementary Table 1. Risk factors associated with serological s in PCOS women**

|  | | **LFS** | **HSI** | **FLI** |
| --- | --- | --- | --- | --- |
| Age | N | 217 | 209 | 83 |
|  | P | 0.719 | 0.533 | 0.024 |
|  | r | -0.025 | 0.043 | 0.248 |
| BMI | N | 209 | 209 | 83 |
|  | P | <0.001 | <0.001 | <0.001 |
|  | r | 0.582 | 0.868 | 0.921 |
| WC | N | 188 | 188 | 83 |
|  | P | <0.001 | <0.001 | <0.001 |
|  | r | 0.548 | 0.730 | 0.922 |
| SBP | N | 81 | 81 | 71 |
|  | P | 0.008 | <0.001 | <0.001 |
|  | r | 0.294 | 0.380 | 0.415 |
| ALT | N | 217 | 209 | 83 |
|  | P | <0.001 | <0.001 | <0.001 |
|  | r | 0.674 | 0.677 | 0.461 |
| AST | N | 217 | 209 | 83 |
|  | P | <0.001 | <0.001 | 0.510 |
|  | r | 0.296 | 0.237 | 0.073 |
| GGT | N | 91 | 91 | 83 |
|  | P | <0.001 | <0.001 | <0.001 |
|  | r | 0.699 | 0.631 | 0.690 |
| ALP | N | 94 | 94 | 83 |
|  | P | 0.007 | 0.004 | 0.015 |
|  | r | 0.277 | 0.292 | 0.267 |
| AST/ALT | N | 217 | 209 | 83 |
|  | P | <0.001 | <0.001 | <0.001 |
|  | r | -0.733 | -0.801 | -0.574 |
| hsCRP | N | 90 | 90 | 80 |
|  | P | <0.001 | <0.001 | <0.001 |
|  | r | 0.479 | 0.651 | 0.676 |
| TG | N | 214 | 206 | 83 |
|  | P | <0.001 | <0.001 | <0.001 |
|  | r | 0.475 | 0.349 | 0.755 |
| HDL-c | N | 212 | 204 | 82 |
|  | P | <0.001 | <0.001 | <0.001 |
|  | r | -0.527 | -0.494 | -0.617 |
| LAP | N | 185 | 185 | 84 |
|  | P | <0.001 | <0.001 | <0.001 |
|  | r | 0.638 | 0.649 | 0.944 |
| 0hGlu | N | 207 | 199 | 77 |
|  | P | <0.001 | <0.001 | <0.001 |
|  | r | 0.390 | 0.305 | 0.495 |
| 2hGlu | N | 216 | 208 | 83 |
|  | P | 0.009 | 0.002 | 0.006 |
|  | r | 0.178 | 0.213 | 0.301 |
| 0hINS | N | 217 | 209 | 83 |
|  | P | <0.001 | <0.001 | <0.001 |
|  | r | 0.878 | 0.584 | 0.713 |
| 2hINS | N | 213 | 205 | 82 |
|  | P | <0.001 | <0.001 | <0.001 |
|  | r | 0.504 | 0.430 | 0.558 |
| HOMA-IR | N | 207 | 199 | 77 |
|  | P | <0.001 | <0.001 | <0.001 |
|  | r | 0.874 | 0.593 | 0.716 |
| QUICKI | N | 207 | 199 | 77 |
|  | P | <0.001 | <0.001 | <0.001 |
|  | r | -0.874 | -0.593 | -0.716 |
| Gutt index | N | 195 | 195 | 76 |
|  | P | <0.001 | <0.001 | <0.001 |
|  | r | -0.493 | -0.423 | -0.578 |
| T | N | 210 | 203 | 80 |
|  | P | 0.358 | 0.621 | 0.889 |
|  | r | 0.064 | 0.035 | 0.016 |
| SHBG | N | 190 | 184 | 79 |
|  | P | <0.001 | <0.001 | <0.001 |
|  | r | -0.477 | -0.539 | -0.686 |
| FAI | N | 187 | 181 | 77 |
|  | P | <0.001 | <0.001 | <0.001 |
|  | r | 0.430 | 0.454 | 0.627 |

**Supplementary table 2. Prevalence of NAFLD in Rotterdam phenotype A to D of PCOS. (N = 188)**

| **Phenotype** | **A**  **N (%)** | **B**  **N (%)** | **C**  **N (%)** | **D**  **N (%)** | **P** |
| --- | --- | --- | --- | --- | --- |
| NAFLD | 49 (36.6%) | 3 (16.7%) | 2 (40.0%) | 8 (25.8%) | 0.222 |

**Supplementary table 3. Prevalence of MAFLD in Rotterdam phenotype A to D of PCOS. (N = 145)**

| **Phenotype** | **A**  **N (%)** | **B**  **N (%)** | **C**  **N (%)** | **D**  **N (%)** | **P** |
| --- | --- | --- | --- | --- | --- |
| MAFLD | 47 (45.6%) | 3 (25.0%) | 2 (40.0%) | 8 (32.0%) | 0.182 |

**Supplementary Table 4. Prevalence of NAFLD in PCOS Patients with Different Characteristics.**

|  | **N** | **Group** | **NAFLD (%)** | **P** |
| --- | --- | --- | --- | --- |
| PCOM | 192 | Yes | 60 (34.5%) | 0.187 |
|  |  | No | 3 (16.7%) |  |
| Hirsutism | 212 | Yes | 28 (40.0%) | 0.192 |
|  |  | No | 44 (31.0%) |  |
| Acne | 213 | Yes | 31 (31.3%) | 0.474 |
|  |  | No | 41 (36.0%) |  |
| Alopecia | 213 | Yes | 25 (33.8%) | 0.997 |
|  |  | No | 47 (33.8%) |  |
| FAI>8 | 187 | Yes | 42 (53.8%) | <0.001 |
|  |  | No | 19 (17.4%) |  |

Notes: Hirsutism was defined as the mFG score ≥5；acne as the IGA score ≥2; and alopecia as the Ludwig score grade I or II or III. The biochemical hyperandrogenism was defined as the 95th percentile of FAI in healthy controls (FAI>8).

**Supplementary Table 5. Pairwise Comparison in 3 Age-Divided Groups.**

|  | **Group 1 vs 2** | **Group 1 vs 3** | **Group 2 vs 3** |
| --- | --- | --- | --- |
| Central obesity | 0.573 | **0.033** | **0.002** |
| Increased blood pressure | 0.326 | **0.034** | 0.239 |
| Hypertriglyceridemia | 0.059 | **0.007** | 0.206 |
| Hyperlipidemia | **0.040** | **0.004** | 0.192 |
| Abnormal blood glucose | 0.978 | 0.254 | 0.161 |
| HOMA-IR≥3.54 | 0.449 | 0.400 | 0.842 |
| Metabolic syndrome | 0.520 | 0.500 | 0.121 |
| Abnormal liver enzymes | 0.324 | 0.525 | 0.772 |
| ALT≥18.2 | 0.984 | 0.849 | 0.800 |
| FAI>8 | 0.835 | 0.081 | 0.983 |
| Hirsutism | 0.368 | 0.089 | 0.289 |
| Acne | 0.828 | 0.114 | 0.032 |
| PCOM | 0.913 | 1.000 | 0.906 |

Notes: Group 1: 18~25 years; Group 2: 26~30 years; Group 3: over 30 years.

**Supplementary Table 6. Risk factors (continuous variables) associated with NAFLD by binary logistic regression in overweight patients.**

| **Continuous variables** | **OR (95% CI)** | **P** |
| --- | --- | --- |
| HOMA-IR | 55.209 (2.717, 1121.799) | 0.009 |
| LAP | 1.064 (0.998, 1.133) | 0.057 |
| ALT | 1.354 (1.020, 1.796) | 0.036 |

**Supplementary Table 7. Risk factors (categorical variables) associated with NAFLD by binary logistic regression in overweight patients.**

| **Categorical variables** | **OR (95% CI)** | **P** |
| --- | --- | --- |
| HOMA-IR≥3.21 | 98.738 (18.319, 532.189) | <0.001 |
| LAP≥41.43 | 8.861 (1.576, 49.824) | 0.013 |

**Supplementary Table 8. Risk factors (continuous variables) associated with NAFLD by binary logistic regression in non-overweight patients.**

| **Continuous variables** | **OR (95% CI)** | **P** |
| --- | --- | --- |
| HOMA-IR | 59.936 (0.622, 5773.594) | 0.079 |
| ALT | 1.293 (0.982, 1.702) | 0.067 |

**Supplementary Table 9. Risk factors (categorical variables) associated with NAFLD by binary logistic regression in non-overweight patients.**

| **Categorical variables** | **OR (95% CI)** | **P** |
| --- | --- | --- |
| HOMA-IR≥2.88 | 110.852 (10.596, 1159.644) | <0.001 |
| ALT≥18.4 | 9.366 (1.313, 66.794) | 0.026 |
